# Multi‐Chamber Intracardiac Thrombi Complicated With Pulmonary and Lower Extremity Thrombosis in Male Patient: Case Report and Literature Reviews

**DOI:** 10.1002/ccr3.71833

**Published:** 2026-01-12

**Authors:** Eba Fufa, Yitagesu Getachew, Samson Mulugeta, Abera Birhanu, Abel Yirga, Amdemeskel Mersha, Banchiaymolu Damtie, Balew Arega, Begashaw Belay, Brook Fanuel, Misgana Lukas

**Affiliations:** ^1^ Department of Internal Medicine Yekatit 12 Hospital Medical College Addis Ababa Ethiopia

**Keywords:** cardiomyopathy, embolism, Ethiopia, sinus rhythm, thrombus

## Abstract

Multi‐chamber intracardiac thrombi are a rare and high‐risk complication in dilated cardiomyopathy, even during sinus rhythm. This case highlights the associated dangers of hemodynamic compromise and systemic embolization. Management requires vigilant anticoagulation and close monitoring to balance thromboembolic and bleeding risks.

## 1 Introduction

Intracardiac thrombosis is a blood clot in the heart that may occur de novo in any of the four cardiac chambers, including atrial appendages [1]. The underlying mechanisms for intracardiac thrombus formation have been attributed to either blood stasis or hematological abnormalities causing hypercoagulability [2]. Multi‐chamber intracardiac thrombi can develop among severe heart failure (HF) patients who are at risk of static blood flow, endothelial dysfunction, and increased hypercoagulable status [3]. Patients with dilated cardiomyopathy, myocardial infarction, and peripartum cardiomyopathy are at risk of developing intracardiac thrombosis [4], but it is extremely rare with sinus rhythm [5]. Coexistent thrombi in the right ventricle (RV) and right atrium (RA) can rarely be found among patients with left ventricle (LV) thrombus. Such patients have an increased risk of systemic and pulmonary embolism [6].

Vitamin K antagonists (VKAs), predominantly warfarin, have traditionally been used and recommended for the prevention and treatment of LV thrombus, but there is a growing interest in replacing VKAs with direct oral anticoagulants (DOACs) such as apixaban, rivaroxaban, edoxaban, and dabigatran [1]. There are a few anecdotal reports and retrospective small case series of surgical excision of LV thrombus [7]. On the other hand, the optimal medical or surgical treatment of right heart thrombi remains unclear, with limited experience with catheter‐directed thrombectomy [8, 9]. The therapy of multi‐chambered intracardial thrombi is challenging because these individuals are complicated with hemodynamic derangements, including shock, acute embolic events that can have cerebral effects, or systemic embolic events with increased mortality or morbidity if left untreated [10].

We describe dilated cardiomyopathy with huge tri‐chamber intracardiac thrombi involving LV, RV, and RA, and other site thrombosis, including lower extremity and pulmonary vein thrombosis, of an adult patient who presented to our hospital. This unusual example demonstrates the relevance of blood stasis as the cause of thrombus development in cardiac chambers, as well as a learning case for clinicians in the management of a huge tri‐chamber intracardiac thrombus in resource‐limited settings.

## 2 Case History and Examinations

A 40‐year‐old male Ethiopian farmer presented with a three‐month history of progressive easy fatiguability, a loss of appetite, and epigastric pain associated with significant but unquantified weight loss. Over the past 2 months before admission, he developed a dry intermittent cough that worsened with lying down, exertional shortness of breath (SOB), orthopnea of two pillows, paroxysmal nocturnal dyspnea (PND), and progressive asymmetric lower extremity swelling, more on the right leg.

Since the past 2 weeks before visits to Yekatit 12 Hospital Medical College's adult emergency department, the cough became productive of frothy sputum mixed with blood, which progressed to frank hemoptysis of clotted blood over the course of 1 week. In addition, he had SOB that occurs at rest and sleeps in a semi‐sitting position due to worsening orthopnea. He also complained of intermittent high‐grade fever and decreased urine output, but no color change. Otherwise, he had no known multimorbid illness, no risky behavior like smoking or alcohol drinking, or illicit as well as traditional medications. He was not treated for tuberculosis (TB). His COVID/vaccine status is unknown, but he has no history of flu‐like symptoms. He was married and a farmer from a rural area. He had never been admitted for a similar complaint. He lost his older brother 5 years ago at the age of 45 with a similar disease course, but he was not diagnosed. He died with massive blood coming from his mouth and nose at home, according to his description.

The patient's Glasgow Coma Scale (GCS) was 15 out of 15, and there were no memory, power, or tone abnormalities. The patient was in respiratory distress with an oxygen saturation of 80% with atmospheric air, a respiratory rate of 25 to 28 breaths/min, and a pulse rate of 109–129 beats per minute. He had no record of fever at presentation but had a low blood pressure of 80/50 mmHg with a regular, weak radial pulse.

Chest examination revealed bilateral coarse crepitus over the lower two‐thirds of the posterior chest and decreased air entry over the left lower posterior third. On the cardiovascular examination, jugular venous pressure was raised with no added sound in the pericardial examination. The abdominal examination revealed positive signs of fluid collection with no palpable organ. Abdominopelvic ultrasound showed only minimal ascites. He had grade II pitting edema, and his right and left legs had a three‐centimeter discrepancy.

## 3 Methods

**FIGURE 1:**
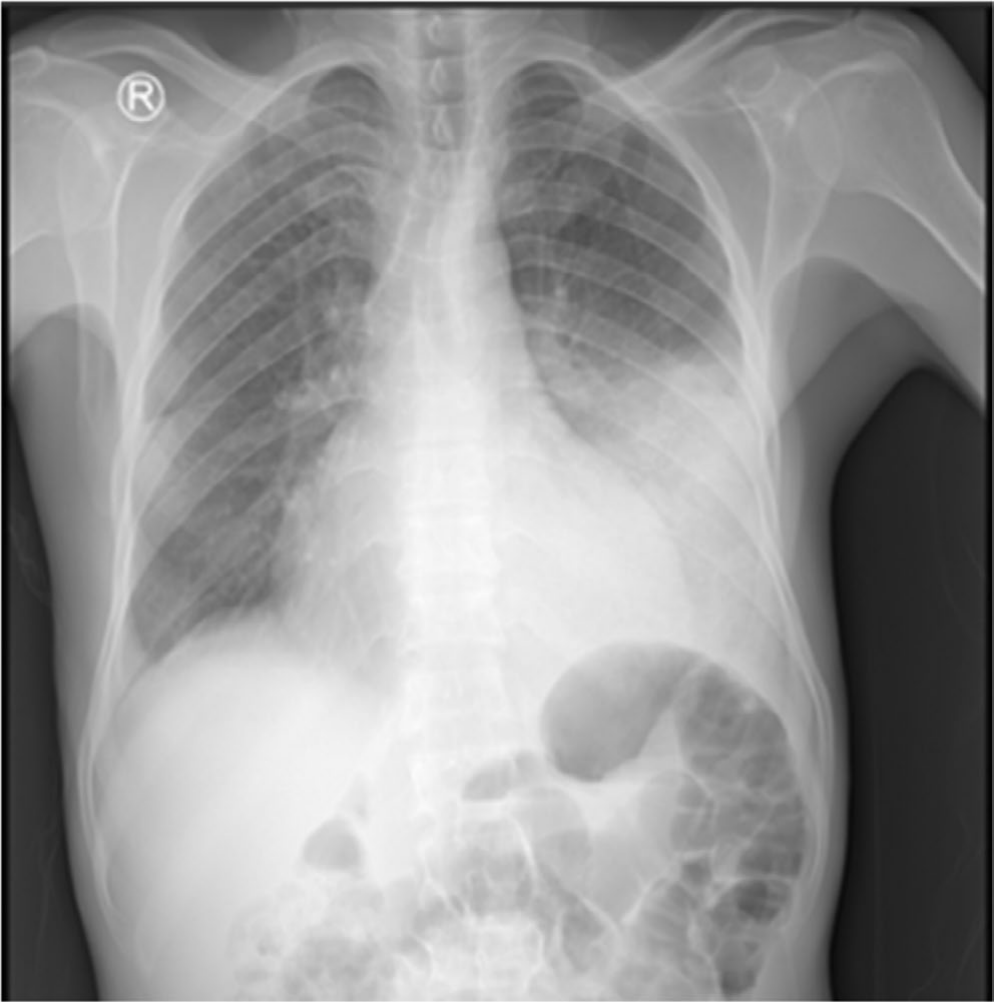
Erect posterior to anterior chest X‐ray showing cardiomegaly with left middle‐lower homogeneous opacity and right side costo‐phrenic angle blunting.

**FIGURE 2:**
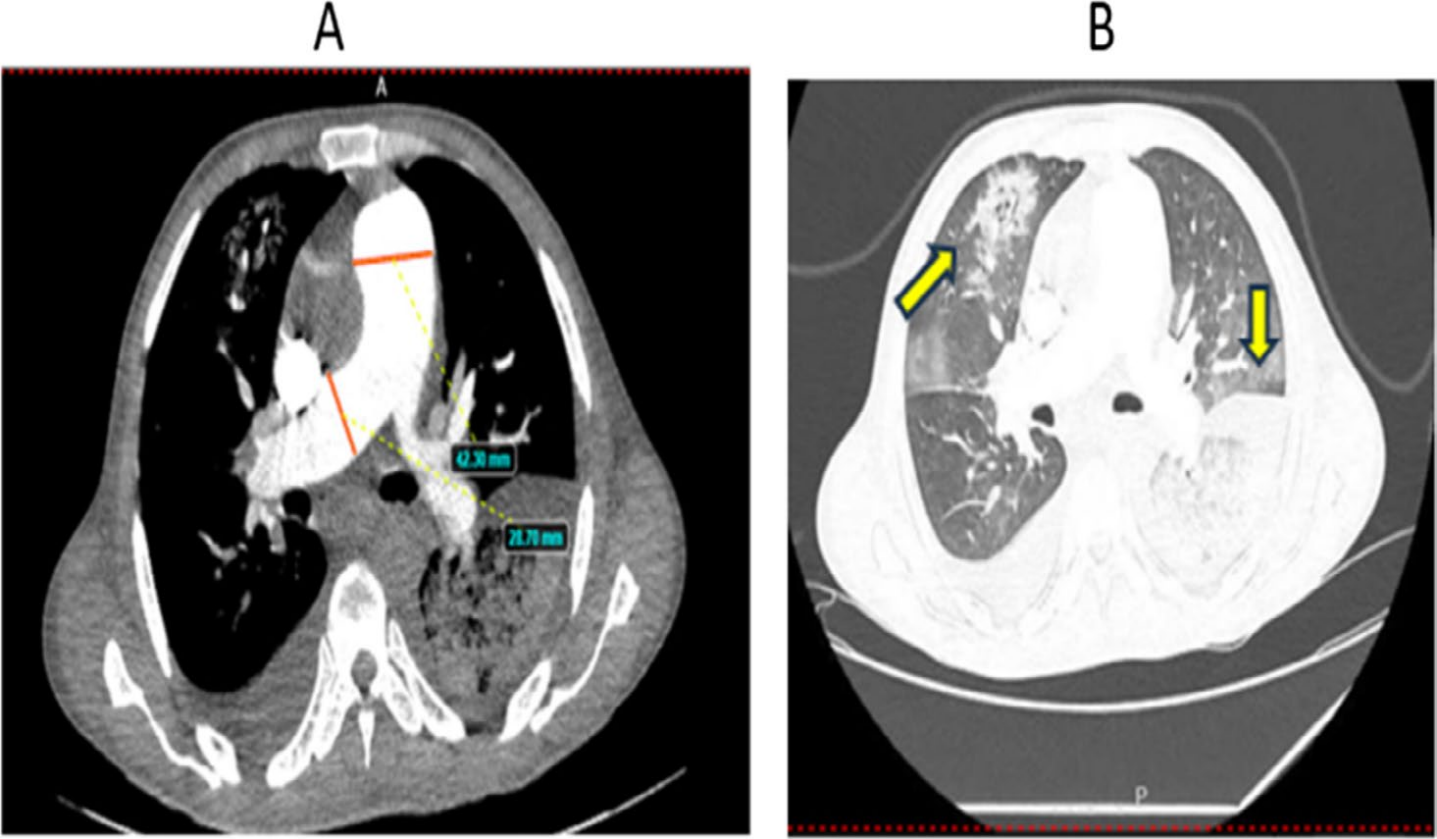
Enlarged bilateral pulmonary arteries and their lobar branches (A) and bilateral lungs with multiple large pulmonary infarctions, the largest at the left lower lobe (B).

Chest X‐ray showed cardiomegaly, left middle, and lower lung field homogenous opacity (Figure 1). Chest computed tomography angiography (CTA) showed enlarged bilateral pulmonary arteries and their lobar branches, and bilateral lungs with multiple large pulmonary infarctions, the largest at the left lower lobe, with bilateral mild pleural effusions (Figure 2A,B).

**FIGURE 3:**
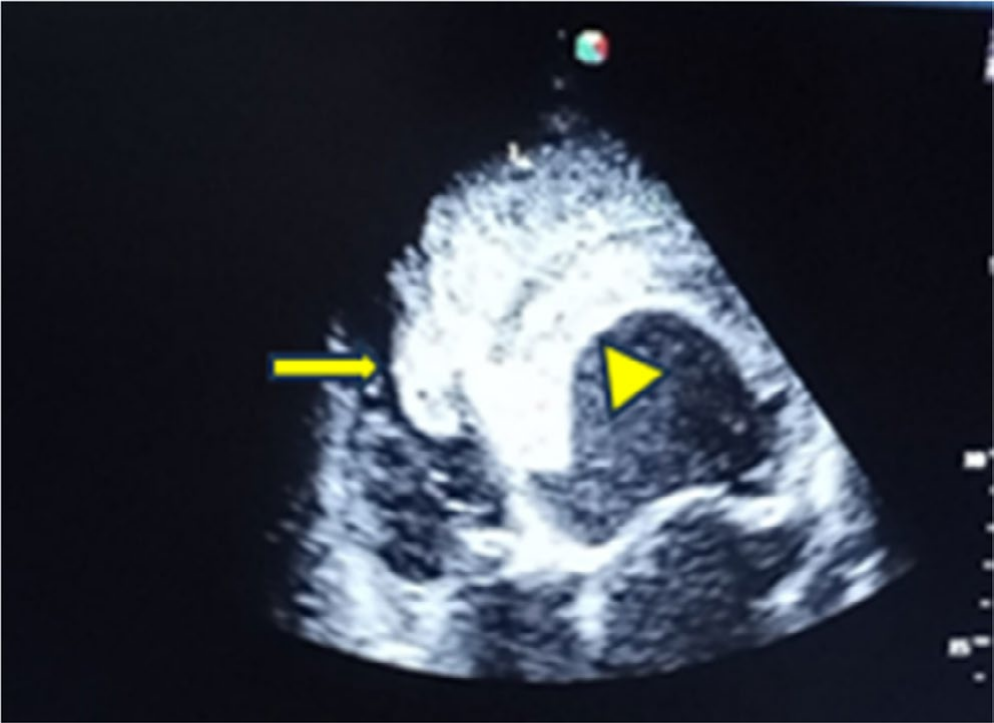
A 2‐dimensional echocardiographic picture showing a large left ventricular thrombus (arrowhead) and a solitary laminar right ventricular thrombus (arrow).

The electrocardiogram showed left axis deviation with poor R‐wave progression. Echocardiography revealed a global LV wall hypokinesis suggestive of non‐ischemic dilated cardiomyopathy with reduced biventricular systolic function (EF of 20% and TAPSE of 13 mm) and secondary mild mitral and tricuspid regurgitation. There is a large left ventricular thrombus (43 by 58 mm) and a solitary laminar right ventricular thrombus (10 × 34 mm) (Figure 3). A transesophageal echocardiogram was not performed due to the patient's critical and unstable condition. Therefore, a patent foramen ovale (PFO) could not be definitively ruled out. The diagnosis of multi‐chamber thrombi was made by transthoracic echocardiogram (TTE), though the images were suboptimal due to the patient's clinical state. Ischemic etiology was excluded based on clinical assessment, a normal ECG without signs of ischemia, and negative serial troponin levels, as coronary angiography was not feasible.

**FIGURE 4:**
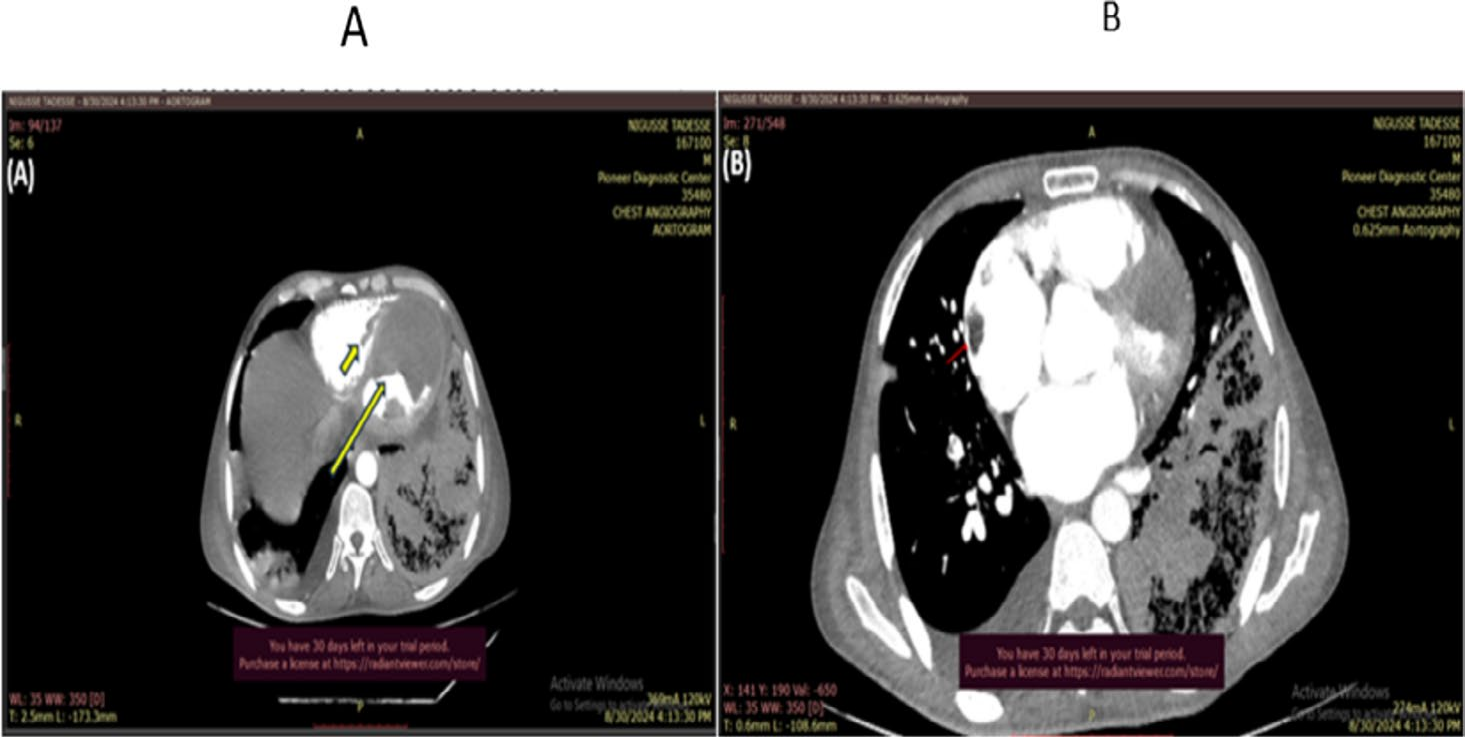
Large LV thrombus (11.5 × 7.5 cm) (long arrow) with markedly dilated LV, RV thrombus (3.6 × 1.6 cm) (short arrow) (A), two right atrial thrombi (2.3 and 1.4 cm) (red arrow) (B).

The cardiac CT scan was not done due to persisting tachycardia, but chest CTA reported a large LV thrombus (11.5 × 7.5 cm) with markedly dilated LV (7.2 cm), RV thrombus (3.6 × 1.6 cm), and two right atrial thrombi (2.3 and 1.4 cm) (Figure 4). Doppler ultrasound of the right lower extremity reported acute proximal deep vein thrombosis. Serial determination of complete blood count and liver enzymes and bilirubin showed a raised value (Table 1).

**TABLE 1:** Serial laboratory determination values of the patient.

Laboratory tests	Done on the day of admission	3 days after admission	Last updated after admission
White blood cell (**×**10^3^/μL)	23.9	16	27.73
Neutrophil (%)	89.3	90.2	89.9
Lymphocyte (%)	4.6	5.1%	5.2
Monocyte (%)	6.1	4.7%	4.9
Red blood cells (**×**10^6^/μL)	4.69	4.15	3.62
Hemoglobin (mg/dL)	13	10.5	9.2
Hematocrit (%)	35.2	30.1	26.4
Mean corpuscular volume (fl)	75	72.5	72.9
Mean corpuscular hemoglobin (pg)	27.7	25.2	25.5
Platelets (**×**10^3^/μL)	168	213	205
Prothrombin time	21	18	21.8
Activated partial thromboplastin time	43.5	36.7	39.4
International normalized ratio	1.76	1.5	1.82
Creatinine (mg/dL)	1.17	0.8	1.3
Blood urea (mg/dL)	32	31	27
Aspartate aminotransferase (μ/L)	223	107	107
Alanine aminotransferase (μ/L)	282	131	146
Alkaline phosphatase (μ/L)	268	383	390
Total bilirubin (mg/dL)	3.33	2.63	3.99
Direct bilirubin (mg/dL)	1	1.14	1.18
Potassium (mEq/L)	2.71	3.29	3.46
Sodium (mEq/dL)	136.51	133	138

Serum albumin was 2.6 g/dL. The coagulation profile is not significantly deranged. Cardiac troponin is normal. The lipid profile showed triglyceride, 51 mg/dL, total cholesterol, 87 mg/dL, high‐density lipoprotein, 22 mg/dL, and low‐density lipoprotein, 30 mg/dL. Lactate dehydrogenase (LDH) level was within normal limits (123 U/L).

## 4 Conclusion and Results

He was admitted to the adult intensive care unit (ICU) with the diagnosis of cardiogenic shock, acute decompensated HF secondary to dilated cardiomyopathy with reduced ejection fraction precipitated by severe community‐acquired pneumonia, tri‐chamber intracardiac thrombi, acute bilateral pulmonary thromboembolism, and right leg proximal acute deep vein thrombosis. He was started on (noradrenaline) standard doses of potent antibiotics (vancomycin and cefepime), hydrocortisone, unfractionated heparin infusion, intravenous furosemide, spironolactone, gastrointestinal prophylaxis, analgesics, and low‐dose morphine for cough suppression. Following a multidisciplinary review, fibrinolysis was contraindicated due to the high risk of catastrophic embolization from mobile ventricular thrombi and the significant hemorrhage risk from the patient's coagulopathy.

After 5 days of admission, he developed hematemesis from multiple episodes of upper gastrointestinal bleeding. Medical management was instituted, and unfractionated heparin was held. On serial determination, there was no significant change in the coagulation profile. Unfortunately, he succumbed to cardiac arrest 24 h after the onset of hematemesis. Endoscopic evaluation and intervention were not possible as he was not hemodynamically stable enough to be moved out of the ICU.

## 5 Discussion

This report details an adult man's case of non‐ischemic dilated cardiomyopathy with cardiogenic shock, tri‐chamber huge intracardiac thrombi, pulmonary thromboembolism, and left leg deep vein thrombosis. The patient was evaluated with echocardiography and CT angiogram, electrocardiography, cardiac biomarkers, and additional laboratory testing.

Similar to our findings, previously thrombi involving the LV, RV, and RA were simultaneously reported among case reports of patients with dilated CMP [6]. Other case reports documented the occurrence of thrombi in two atria and the LV [11, 12], the two ventricles and LA [12, 13], and involving all cardiac chambers [13, 14, 15, 16]. Premortem diagnosis of thrombi in three cardiac chambers at the same time in patients with pulmonary emboli is extremely rare, and we are aware of only a few case reports. In agreement with our finding, two other case reports documented bilateral PTE and DVT in addition to multiple intracardiac thrombi [12, 16]. The outcome of patients complicated with pulmonary embolism and multiple chamber thrombosis on the background of advanced HF is expected to be poor. Our patient developed cardiogenic shock, and despite appropriate management with the involvement of experienced cardiologists, he died in the hospital. Similarly, two case reports documented the in‐hospital mortality of patients diagnosed with multiple cardiac chamber thrombi [2, 15]. The distribution of intracardiac thrombi, chamber involved, treatment outcomes, and clinical presentation are presented in Table 2.

**TABLE 2:** Review of case reports documenting three or more heart‐chambered (at least three and above) thrombi in adult patients.

Studies	Sex/age	Chamber involved	Others site thrombosis	Diagnosis and comorbidities	Management (anticoagulant)	Treatment outcome
Ida et al. (2014) [2]	M/55	All chambers	PTE	Ischemic CMP, atrial flatter, PFO	LMWH and mechanical thrombectomy	Died in the hospital
Singh et al. (2015) [10]	M/48	All chambers	No other sites of thrombosis	Alcoholic CMP	Anticoagulated^a^	Survived
Elikowski et al. (2020) [11]	M/60	RV, RA, LV	PTE	Dilated CMP	Apixaban	Survived
Valle et al. (2015) [12]	F/22	RV, LV, LA	PTE	Peripartum dilated CMP	Warfarin and thrombi resolved	Survived
Agac et al. (2011) [13]	M/22	All chambers	No other sites of thrombosis	Acute myocarditis	Not mentioned	Died in the hospital
Kunkler et al. (2013) [14]	M/74	RA, LA, RV	Bilateral PTE, DVT	CAD, HPN, PFO	UFH with Warfarin	Survived
Izaga et al. (2020) [11]	M/47	All chambers	Bilateral PTE, DVT	PFO	UFH, embolectomy, and PFO closure	Survived
Nishigawa et al. (2011) [15]	M/74	LA, RA, LV	No other sites of thrombosis	HPN, Atrial fibrillation	Cardiopulmonary bypass and right atrial atriotomy, UFH	Survived
Shah et al. (2022) [16]	F/27	LA, LV, RV	DVT	COVID‐19 and DCP		Survived

Abbreviations: CAD, coronary arterial diseases; CMP, cardiomyopathy; COVID‐19, coronavirus disease 2019; DVT, deep vein thrombosis; F, female; HTN, hypertension; LA, left atrium; LMWH, low‐molecular‐weight heparin; LV, left ventricle; M, male; PFO, patent foramen ovale; PTE, pulmonary thromboembolism; RA, right atrium; RV, right ventricle; UFH, unfractionated heparin.

^a^ Specific anticoagulant not mentioned.

Veins of the lower extremities are classic sources of pulmonary embolisms and the thrombi in the right heart chambers. However, in the presence of PFO, the thrombus had migrated from the IVC and also migrated to the left heart. In our patient, there is no evidence of a patent foramen ovale on echocardiography and chest CTA assessment. In our patient, the source of the right chamber thrombi and thereby pulmonary embolism might have originated from the DVT in the right leg, which likely formed as a result of prolonged immobility from his severe heart illness.

On arrival at the hospital, the patient was hemodynamically unstable in cardiogenic shock, and a multidisciplinary team opted for conservative management with unfractionated heparin, management of the shock, and heart failure. We cannot further investigate the patient for possible other hypercoagulable factors, including proteins C and S, because there are signs of liver injury affecting the reliability of the finding.

In conclusion, timely identification and management of intracardiac thrombus in cardiomyopathy is of immense importance, as this condition may be fatal. Management in such uncommon cases remains challenging, and the complete scenario should always be taken into consideration. A multidisciplinary consensual approach should be encouraged. Emergency surgical management, with systemic anticoagulation, might save the lives of patients.

## Author Contributions

**Eba Fufa:** conceptualization, data curation, formal analysis, funding acquisition, investigation, methodology, project administration, resources, software, supervision, validation. **Yitagesu Getachew:** conceptualization, data curation, formal analysis. **Samson Mulugeta:** conceptualization, data curation, formal analysis, methodology. **Abera Birhanu:** conceptualization, data curation, formal analysis, methodology. **Abel Yirga:** conceptualization, data curation, investigation, methodology. **Amdemeskel Mersha:** conceptualization, data curation, writing – original draft. **Banchiaymolu Damtie:** conceptualization, data curation, investigation. **Balew Arega:** conceptualization, data curation, formal analysis, investigation, writing – original draft, writing – review and editing. **Begashaw Belay:** conceptualization, data curation, investigation, methodology. **Brook Fanuel:** conceptualization, data curation, visualization, writing – original draft. **Misgana Lukas:** conceptualization, data curation, formal analysis, investigation, methodology.

## Funding

The authors have nothing to report.

## Ethics Statement

The authors have nothing to report.

## Consent

Written informed consent was obtained from the patient to publish this report in accordance with the journal's patient consent policy.

## Conflicts of Interest

The authors declare no conflicts of interest.

## Data Availability

The authors have nothing to report.
